# Effect of Different Ratios of Yinchen and Gancao Decoction on ANIT-Treated Cholestatic Liver Injury in Mice and Its Potential Underlying Mechanism

**DOI:** 10.3389/fphar.2021.611610

**Published:** 2021-04-15

**Authors:** Huizong Su, Qian Wang, Yue Li, Jingyi Jin, Bo Tan, Dongming Yan, Bin Zou, Guochao Song, Fengyi Weng, Furong Qiu

**Affiliations:** Clinical Pharmacokinetic Laboratory, Shuguang Hospital Affiliated to Shanghai University of Traditional Chinese Medicine, Shanghai, China

**Keywords:** cholestatic liver injury, toll-like receptor 4, nuclear factor kappa B, cytokines and chemokines, Yinchen and Gancao decoction

## Abstract

Cholestasis is a pathological state that leads to serious liver disease; however, therapeutic options remain limited. Yinchen and Gancao are often used in combination at different ratios in traditional Chinese formulae for the treatment of jaundice and cholestasis. In the present study, we investigated the effect of decoctions containing different ratios of Yinchen and Gancao (YGD) on alpha-naphthyl isothiocyanate (ANIT)-treated intrahepatic cholestasis (IC) in mice, and further explored the underlying mechanism. Treatment with 0:4 and 1:4 YGD significantly reduced plasma total bile acid (TBA), total bilirubin (TBIL), aspartate aminotransferase (AST), alanine aminotransferase (ALT), and alkaline phosphatase (ALP) activities; decreased unconjugated and conjugated bile acid levels; and improved hepatocyte necrosis and inflammatory cells recruitment to hepatic sinusoids. Moreover, the expression levels of Toll-like receptor 4 (TLR4), interleukin-1β (IL-1β), IL-6, tumor necrosis factor alpha (TNF-α), C-C ligand 2 (CCL2), and C-X-C ligand 2 (CXCL2) in the liver were significantly reduced. However, treatment with 4:1 and 4:0 YGD increased plasma TBA, TBIL, AST, ALT, and ALP activities and aggravated liver cell injury and inflammation. Moreover, the mRNA expression of the bile salt export pump (BSEP) in the liver was significantly increased in mice treated with 4:0 YGD. The present study demonstrates that YGD containing a high proportion of Gancao, which inhibits the TLR4/NF-κB pathway and reduces the inflammatory response, had protective effects against ANIT-treated IC in mice. However, YGD containing a high proportion of Yinchen aggravated the ANIT-treated IC in mice, which may be related to upregulation of BSEP and boosting bile acid regurgitation from damage cholangiocytes to liver in ANIT-treated IC mice.

## Introduction

Cholestatic liver injury is a clinical syndrome resulting from the impairment of bile formation and/or bile flow coupled with the intracellular retention of toxic bile constituents, including bile acids and salts (Hirschfield et al., 2013). Cholestasis may mainly develop from autoimmune, infectious, drug-induced, metabolic, or genetic disorders (Boyer, 2007; Hirschfield et al., 2013). Without appropriate treatment, cholestasis ultimately leads to hepatic fibrosis, cirrhosis, and even liver failure (Abshagen et al., 2015). Cholestatic liver injury is assumed to be caused by bile acids accumulation in the liver, which lead to toxicity. Recent studies suggest that cholestatic liver injury is related to the recruitment of inflammatory cells including neutrophils and monocytes to the liver, which kills hepatocytes *via* the release of potent reactive oxygen species (ROS). The TLR4/NF-κB signaling pathway is involved in the recruitment of inflammatory cells to hepatic sinusoids and the sites of liver injury (Cai et al., 2017; Li et al., 2017); thus, inhibition of this pathway reduces the recruitment of neutrophils and monocytes to areas of injury, showing a protective effect against cholestatic liver injury (Li et al., 2017). Ursodeoxycholic acid (UDCA) and obeticholic acid (OCA) are the only two drugs approved by the U.S. Food and Drug Administration for the treatment of cholestasis. However, both UDCA and OCA have a limited therapeutic effect in primary sclerosing cholangitis (PSC) or late-stage primary biliary cholangitis (PBC) (de Vries and beuers, 2017). Thus, the development of novel drugs for the treatment of cholestasis is necessary.

According to the mechanism of cholestatic liver injury, therapeutic strategy in treatment of cholestasis should reduce formation of inflammatory cytokines and chemokines and infiltration of inflammatory cells to liver *via* inhibition of the TLR4/NF-κB pathway, in addition to reducing hepatic accumulation of bile acids.

Alpha-naphthyl isothiocyanate (ANIT) is a hepatotoxin experimentally used in rodents to model human intrahepatic cholestasis (Kossor et al., 1995). Administration of a single dose of ANIT can cause cholestasis and liver injury in mice, as characterized by cholangiocytes damage, bile duct obstruction, and infiltration of inflammatory cells around sinusoids (Desmet et al., 1968; Cullen et al., 2016). Previous studies have revealed that inflammation significantly contributes to ANIT-treated hepatotoxicity (Faiola et al., 2006; Kodali et al., 2006; Cullen et al., 2016). Our previous findings showed that UDCA aggravates liver injury in ANIT-treated model mice (Zhang et al., 2018). For over a thousand years, Yinchen (the aerial part of *Artemisia capillaris* Thunb., *Artemisiae Scopariae* Herba) has been used to treat jaundice due toMa its choleretic effect (Wu et al., 2019), while Gancao (the honeyed root and rhizome of *Glycyrrhizauralensis* Fisch., Glycyrrhizae Radix et Rhizoma) has been administered for the treatment of liver diseases, such as liver fibrosis, hepatitis, and drug-induced liver injury (Yang et al., 2017). Gu et al. (2014), Ma et al. (2018) found that glycyrrhizin, which is the main component of Gancao, displays anti-inflammatory activity by inhibiting the TLR4/NF-κB signaling pathway. Moreover, chlorogenic acid, which is the main component of Yinchen, has been shown to protect against Con A-induced hepatitis in mice by inhibiting the same signaling pathway (Yuan et al., 2017). Yinchen and Gancao are often used in combination for the treatment of jaundice and cholestasis; however, their ratios vary in different formulae such as Yinchensini decoction (Yinchen:Gancao 1:1), Yinchenzhufu decoction (Yinchen:Gancao 3:1), and JiaweiYinchenzhufu decoction (Yinchen:Gancao 3–6:1), etc (Yan et al., 2010; Meng and Wang, 2011; Mao et al., 2015; Lai et al., 2018). The present study aimed to elucidate the anti-inflammatory mechanism of decoctions containing different ratios of Yinchen and Gancao in the treatment of cholestatic liver injury. This information could be provide new insights into the effect of YGD in the treatment of cholestasis.

## Materials and Methods

### Drugs and Reagents

Gancao (*Glycyrrhizae Radix et Rhizoma*, Lot: 171124) and Yinchen (*Artemisiae Scopariae Herba*, Lot: 170726) were purchased from Shanghai Kangqiao Herbal pieces Co. Ltd. (Shanghai, China) and authenticated by Professor Li Liu from the Laboratory of Traditional Chinese Medicine (TCM) Preparation, Shuguang Hospital affiliated to Shanghai University of TCM. The glycyrrhizic acid content of Gancao decoction and the chlorogenic acid content of Yinchen decoction was 14.93 mg/g and 1.78 mg/g, respectively, which was determined by high-performance liquid chromatography (HPLC). The following reference standards were purchased from Sigma−Aldrich (St. Louis, MO, United States): chenodeoxycholic acid (CDCA), deoxycholic acid (DCA), cholic acid (CA), taurocholic acid (TCA), taurodeoxycholic acid (TDCA), taurochenodeoxycholic acid (TCDCA), tauromuricholic acid (TMCA), 2,2,3,4,4-5 deuterated lithocholic acid (d5-LCA), and glycyrrhetinic acid (GA). The reference standards of β-muricholic acid (β-MCA) and α-muricholic acid (α-MCA), were purchased from TRC (Toronto, Canada). Glycyrrhizic acid and chlorogenic acid standards were supplied by the National Institutes for Food and Drug Control (Beijing, China). The purity of each standard was ≥98%. α-Naphthyl isothiocyanate (ANIT) was obtained from Sigma−Aldrich (St. Louis, MO, United States). Antibodies against TLR4, MyD88, p-NF-κB-p65, and β-actin were supplied by Santa Cruz Biotechnology (CA, United States), and IL-1β, IL-6, CCL2, and TNF-α ELISA kits were obtained from Anogen (Ontario, Canada). The RNAiso Plus reagent and Primescript™ RT reagent kits were obtained from Takara Bio (Otsu, Japan), and the qPCR Master Mix was purchased from Yeasen Biotech Co. Ltd. (Shanghai, China).

### Preparation of Yinchen and Gancao Decoction

A mass of 30.0 g Yinchen was placed into a round-bottomed flask containing 450 ml distilled water and boiled at 100°C for 30 min. After filtration, the residue was extracted using 450 ml distilled water. Subsequently, a mass of 30.0 g licorice was placed into a round-bottomed flask containing 300 ml distilled water and boiled at 100°C for 30 min. After filtration, the residue was extracted using 450 ml distilled water. The Yinchen extract was added to the Licorice extract to produce different ratios of YGD (Yinchen:Gancao: 4:0, 0:4, 4:1, 2:1, 1:1, 1:2, 1:4). The prepared YGDs were stored at 4°C for use in subsequent animal experiments.

### Animal Experiments and Sample Collection

Male C57BL/6 mice (20 ± 2 g; 4-week-old) were purchased from Shanghai Slack Laboratory Animal Co., Ltd. (Shanghai, China). Mice were housed at 25 ± 2°C and relative humidity of 60–70% under a 12/12 h light/dark cycle. Mice were provided free access to standard diet and tap water and allowed to acclimatize for 1 week prior to the experiment. The animal experiments were conducted according to the Institutional Guide for the Care and Use of Laboratory Animals and were approved by the Institutional Committee of Shanghai University of TCM under the ethical code PZSHUTCM190322006. Mice were randomly divided into nine groups (n = 6 per group): (A) normal; (B) ANIT; (C) ANIT + YGD (4:0); (D) ANIT + YGD (0:4); (E) ANIT + YGD (4:1); (F) ANIT + YGD (2:1); (G) ANIT + YGD (1:1); (H) ANIT + YGD (1:2); and (I) ANIT + YGD (1:4). The body weight were measured once per day. Mice were administered normal saline or YGD (i.g.) 10 ml/kg for 10 days. On day 7, 4 h after administration of normal saline or YGD, all groups (except the normal group, which received an equal volume of olive oil) received i.g. treatment with ANIT (75 mg/kg) dissolved in olive oil. Mice were fasted for 12 h following the last dose, anesthetized with pentobarbital sodium (65 mg/kg, i.p.), and subsequently euthanized for collection of the blood, liver, and gallbladder, which were stored until further analysis.

### Biochemical Assay

Mouse plasma was tested for alkaline phosphatase (ALP) activity, aspartate aminotransferase (AST) activity, alanine aminotransferase (ALT) activity, total bilirubin (TBIL), and total bile acid (TBA) concentration using a commercially available clinical testing kit and a biochemical analysis system (Beckman Coulter, AU5800, Germany).

### Histological Examination

Liver samples were fixed in 4% paraformaldehyde, embedded in paraffin, and cut into 5-μm slices. The sections were subsequently stained with H&E for the evaluation of necrosis. Slides were examined under an Olympus BX41 microscope at a magnification of ×40 and ×200.

### Western Blotting

Total protein was extracted from the liver tissue (Tu et al., 2012), separated by 8% sodium dodecyl sulfate polyacrylamide gel electrophoresis (SDS-PAGE), and transferred to PVDF membrane. After blocking in 5% skim milk (Sango Biotech, Shanghai, China) for 1 h at room temperature, the membranes were further probed with specific antibodies against TLR4, MyD88, p-NF-κBp65, and β-actin overnight at 4°C. After washing three times with TBST, the membranes were incubated with the appropriate secondary antibody for 1 h at room temperature. A Bio-Rad Imaging System (Bio-Rad, Hercules, CA, United States) was employed to detect protein bands using ECL reagent (Beyotime Biotech, Shanghai, China). β-Actin was used as a loading control, and the intensities of the protein bands were quantitated using the ImageJ software (National Institutes of Health, Bethesda, MD, United States).

### ELISA

The concentrations of TNF-α, IL-6, IL-1β, CCL2, and CXCL2 in the incubation medium or mouse liver tissues were assessed by an enzyme-linked immunosorbent assay (ELISA) according to the manufacturer’s instructions (Bio-Swamp Life Science, Shanghai, China). Absorbance (OD) was measured at 450 nm.

### Quantitative Real-Time PCR

Total mRNA from mouse liver tissues was extracted using TRIzol reagent according to the manufacturer's instructions (Sango Biotech, Shanghai, China). cDNA was transcribed using the PrimeScript™ RT Master Mix kit (Takara Bio Inc., Japan) from 500 ng purified total mRNA and amplified in a 20-μl PCR reaction containing specific oligonucleotide primers as shown in Table 1. Forty cycles of amplification were set as follows: denaturation at 95°C for 5 min, annealing at 95°C for 10 s, and extension at 60°C for 30 s. The quantity of mRNA was normalized to murine *GADPH*, and the relative mRNA expression was calculated using 2^−ΔΔct^.

**TABLE 1:** Primer sequences for RT-PCR.

Gene	Forward primer (5′-3′)	Reverse primer (5′-3′)
*Bsep*	GGA​CAA​TGA​TGT​GCT​TGT​GG	CAC​ACA​AAG​CCC​CTA​CCA​GT
*Ccl2*	CCA​GCA​AGA​TGA​TCC​CAA​TGA	TCT​CTT​GAG​CTT​GGT​GAC​AAA​AAC
*Cxcl2*	CCA​ACC​ACC​AGG​CTA​CAG​G	GCG​TCA​CAC​TCA​AGC​TCT​G
*Gapdh*	TGT​GAA​CGG​ATT​TGG​CCG​TA	ACT​GTG​CCG​TTG​AAT​TTG​CC

### Quantitation of Bile Acids by LC-MS/MS

BA extraction was performed according to a previously described method (Zhang et al., 2018). A mass of 100 mg liver tissue was placed in a microcentrifuge tube, to which 900 μl 70% acetonitrile and 5 μl d5-LCA (internal standard, IS, 1.5 g/ml) were added. The mixture was homogenized, vortex-mixed for 5 min, and centrifuged at 10,000 rpm for 10 min, following which the supernatant was transferred to an autosampler for analysis. BAs were determined using an ultra-fast liquid chromatography (UFLC) system (Shimadzu, Japan) and an API 4000 quadrupole linear ion trap hybrid mass spectrometer (QTRAP) coupled to an electrospray ionization (ESI) source (Applied Biosystems, Foster City, CA, United States).

### Statistical Analysis

Data were analyzed using the SPSS 21.0 software and are expressed as the mean ± S.E. Differences between groups were calculated by one-way ANOVA and Dunnett’s test. Differences were considered statistically significant at *p* < 0.05.

## Results

### Effect of YGD on ANIT-Treated Liver Injury in Mice

**FIGURE 1:**
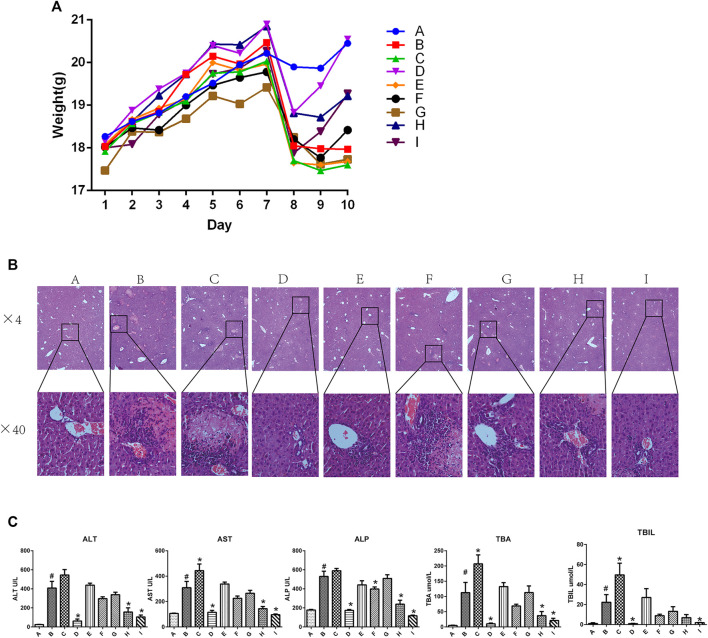
Effect of YGD on ANIT-treated cholestasis model mice **(A)** Changes in body weight in each group of mice during the experiment. **(B)** H&E-stained liver sections from normal and ANIT-treated cholestasis model mice following treatment with different ratios of YGD. **(C)** Plasma levels of biochemical indicators following pretreatment of mice with different ratios of YGD. Normal (A), ANIT (B), ANIT + YGD (4:0) (C), ANIT + YGD (0:4) (D), ANIT + YGD (4:1) (E), ANIT + YGD (2:1) (F), ANIT + YGD (1:1) (G), ANIT + YGD (1:2) (H), and ANIT + YGD (1:4) (I). ^*^ *p* < 0.05 vs. normal group; ^#^ *p* < 0.05 vs. model group.

Figure 1A shows the body weight in each group of mice from day 1 to day 10. The body weight in all groups increased from day 1 to day 7, and sharply decreased on day 7 in all groups except the ANIT group (group B). However, mice in groups D, H, and I rapidly gained weight as compared with those in the group B, and the body weight of mice in group D was restored to near-normal levels by day 10. In contrast, there were no differences among the group B, E, F, and G. Mice in group C had a reduced body weight as compared with that of mice in the group B.

As shown in Figure 1B, inflammatory cells infiltration and hepatocytes necrosis were clearly identified in the group B as compared with those in group A. A significant enlargement of the gallbladder suggested increased bile flow and biliary pressure in the group B, C, E, F, and G (data not shown). Hepatocyte necrosis and bile ductular reaction were ameliorated in group D, H, and I. However, hepatocyte necrosis was slightly mitigated in the group E, F, and G. Unfortunately, liver damage was aggravated in the group C, as reflected by cholangiocytes damage, hepatocytes necrosis, and inflammatory cells infiltration.

As shown in Figure 1C, the activities of plasma ALT, AST, ALP, TBA, and TBIL were significantly elevated 16.3-fold, 2.9-fold, 3.0-fold, 17.3-fold, 22.2-fold, respectively, in ANIT group mice as compared with those in the normal group. Plasma activities of ALT (84.9, 61.7, and 74.3%), AST (62.8, 53.5, and 68.9%), ALP (67.5, 55.0, and 77.9%), and TBA (86.0, 56.2, and 75.0%), and TBIL (95.9, 52.5 and 91.4%) were decreased significantly in the group D, H and I, respectively, compared with those in ANIT group. In contrast, there was no significant change in the plasma biochemical markers in the group E, F and G. However, these plasma biochemical markers increased significantly in the group C compared with those in ANIT group (*p* < 0.05).

### Effect of YGD on Hepatic Bile Acid Levels in ANIT-Treated Model Mice

**FIGURE 2:**
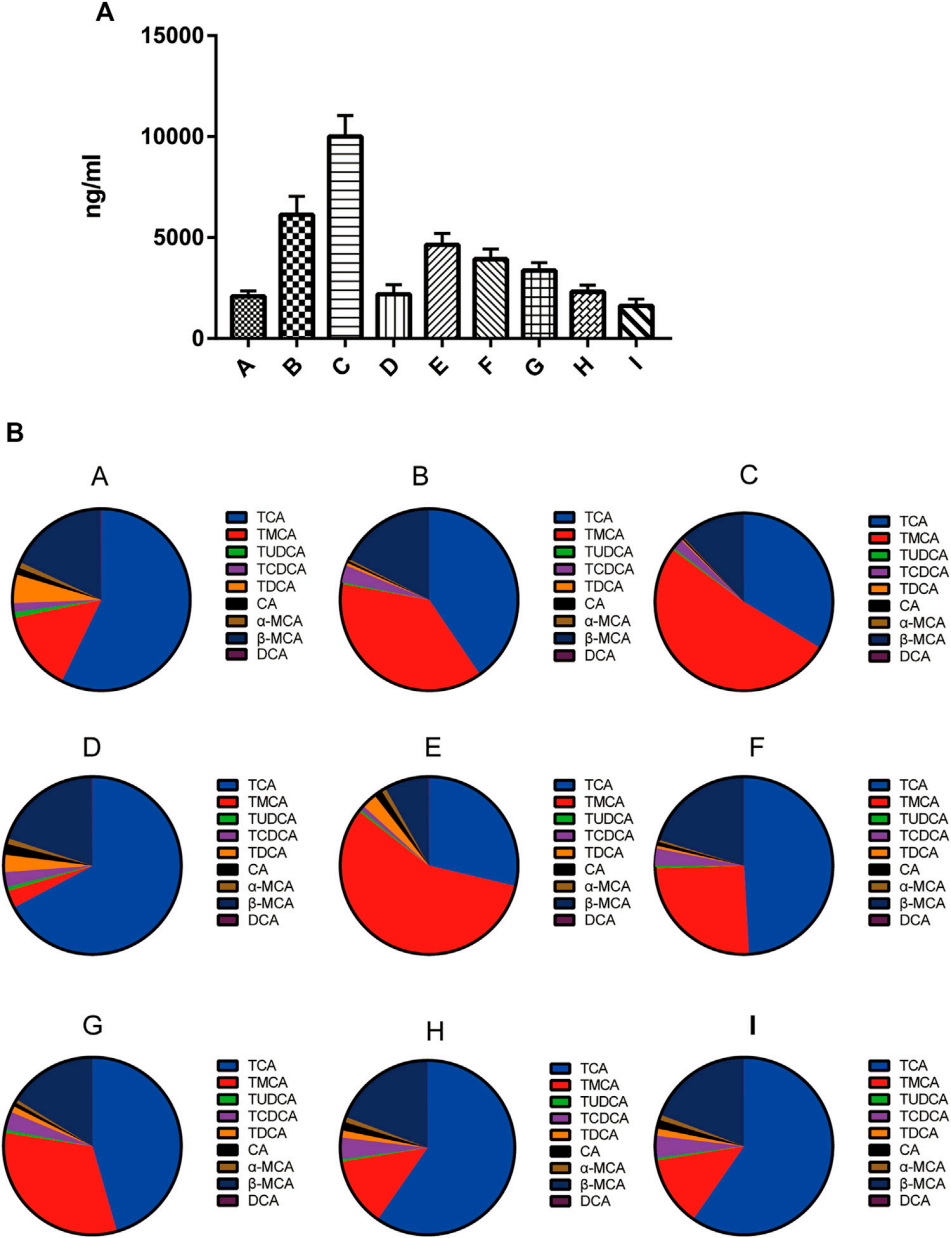
Effect of YGD on the BA composition of the liver **(A)** The total bile acids from normal and ANIT-treated cholestasis model mice following pretreatment with different ratios of YGD. **(B)** The composition of bile acids from normal and ANIT-treated cholestasis model mice following pretreatment with different ratios of YGD. Normal **(A)**, ANIT (B), ANIT + YGD (4:0) (C), ANIT + YGD (0:4) (D), ANIT + YGD (4:1) (E), ANIT + YGD (2:1) (F), ANIT + YGD (1:1) (G), ANIT + YGD (1:2) (H), and ANIT + YGD (1:4) (I). ^*^ *p* < 0.05 vs. normal group; ^#^ *p* < 0.05 vs. model group.

As shown in Figure 2A, the level of total bile acids (TBA) in the mice livers of the group B was 2.93-fold higher than that of the group A. In comparison with the group B, the TBA level in groups D, H, and I was decreased by 64, 62, and 74%, respectively. In contrast, the TBA level in group C was increased by 63%. As shown in Figures 2A,B the liver TBA level was the lowest in normal group mice, with the major liver BAs being TCA (60.0%) and β-MCA (15.0%) followed by taurine-conjugated TMCA (14.1%). The composition of liver BAs was changed in mice of the group B, with TCA, TMCA, β-MCA and TCDCA by 40.6, 37.8, 17.6, and 3.0%, respectively. Pretreatment with 0:4, 1:2, or 1:4 YGD restored the levels of TCA and TMCA in the liver, with TCA being reduced by 75, 40, and 56%, and TMCA by 98, 67, and 93%, respectively. However, 4:0 YGD increased the levels of TMCA and TCA by 123 and 36%, respectively.

The BA composition was similar in the group A, D, H, and I, the latter of which showed a significant decrease in TMCA. The BA composition was similar in the group B, C, E, F, and G. These results indicate that treatment with 0:4 YGD reduced bile acids accumulation in the liver resulted from ANIT treatment but pretreatment with 4:0 and 4:1 YGD aggravated bile acids accumulation in the liver.

### Effect of YGD on the Release of Inflammatory Cytokines and Chemokines Mediated by the TLR/NF-κB Signaling Pathway

**FIGURE 3:**
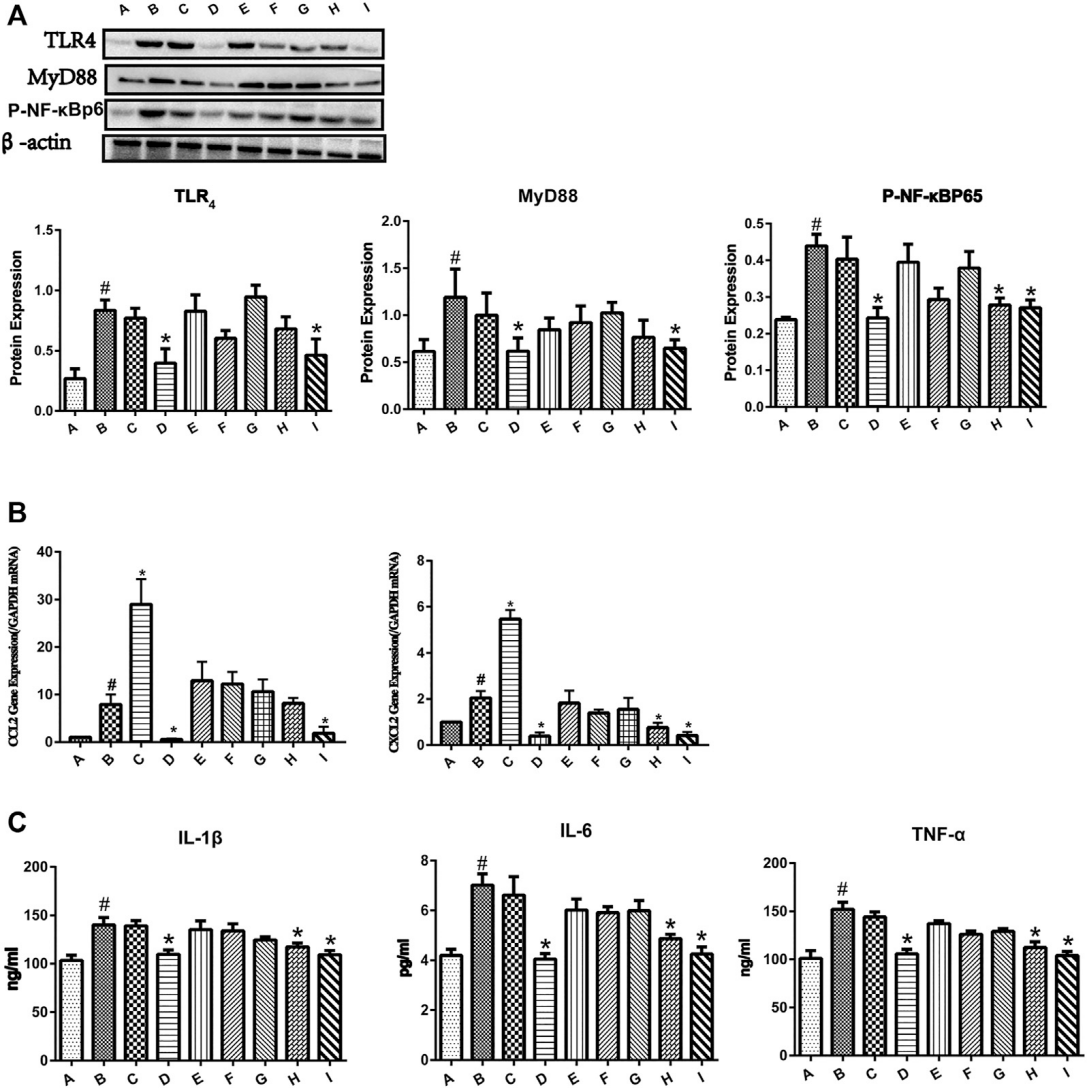
Effect of YGD on the TLR4/NF-κB signaling pathway in ANIT-treated cholestasis model mice. **(A)** The protein expression levels of TLR4, MYD88, and p-NF-κBp65 in mouse liver were determined by western blotting. **(B)** The mRNA expression levels of CCL2 and CXCL2 were determined by RT-PCR. **(C)** The protein expression levels of IL-1β, IL-6, and TNF-α were determined by ELISA. Normal (A), ANIT (B), ANIT + YGD (4:0) (C), ANIT + YGD (0:4) (D), ANIT + YGD (4:1) (E), ANIT + YGD (2:1) (F), ANIT + YGD (1:1) (G), ANIT + YGD (1:2) (H), and ANIT + YGD (1:4) (I). ^*^ *p* < 0.05 vs. normal group; ^#^ *p* < 0.05 vs. model group.

As shown in Figure 3A, the protein expressions of TLR4, MyD88, and p-NF-κBp65 were significantly increased in the group B. Pretreatment with 0:4 or 1:4 YGD showed significant inhibition of TLR4, MyD88, and p-NF-κBp65 protein expression as compared with those in the group B. The phosphorylation of p65 was increased in the ANIT group, and this effect was reversed following treatment with 1:2 YGD (*p* < 0.05).

As shown in Figure 3B, the mRNA expression levels of CCL2 and CXCL2 were significantly increased in the ANIT group. Pretreatment with 0:4 or 1:4 YGD significantly reversed the ANIT-treated increase in the chemokine CCL2 by 93 and 77%, respectively. Although the expression of CCL2 in the group H was inhibited, the difference was not statistically significant. Administration of 0:4, 1:2, or 1:4 YGD significantly reversed the ANIT-treated increase in the chemokine CXCL2 by 81, 63, and 80%, respectively.

As shown in Figure 3C, the levels of IL-1β, IL-6, and TNF-α in the ANIT group were significantly higher than those in the normal group. Administration of 0:4, 1:2, or 1:4 YGD significantly reduced the levels of these proinflammatory cytokines as compared with those in the ANIT group. In contrast, there was no significant inhibition of TLR4, MyD88, or p-NF-κBp65 protein expression or the levels of inflammatory cytokines and chemokines following treatment with 4:1, 2:1, or 1:1 YGD. Unfortunately, treatment with 4.0 YGD increased the levels of CCL2 and CXCL2 to 266 and 167%, respectively.

### Effect of YGD on BSEP Expression in Mice

**FIGURE 4:**
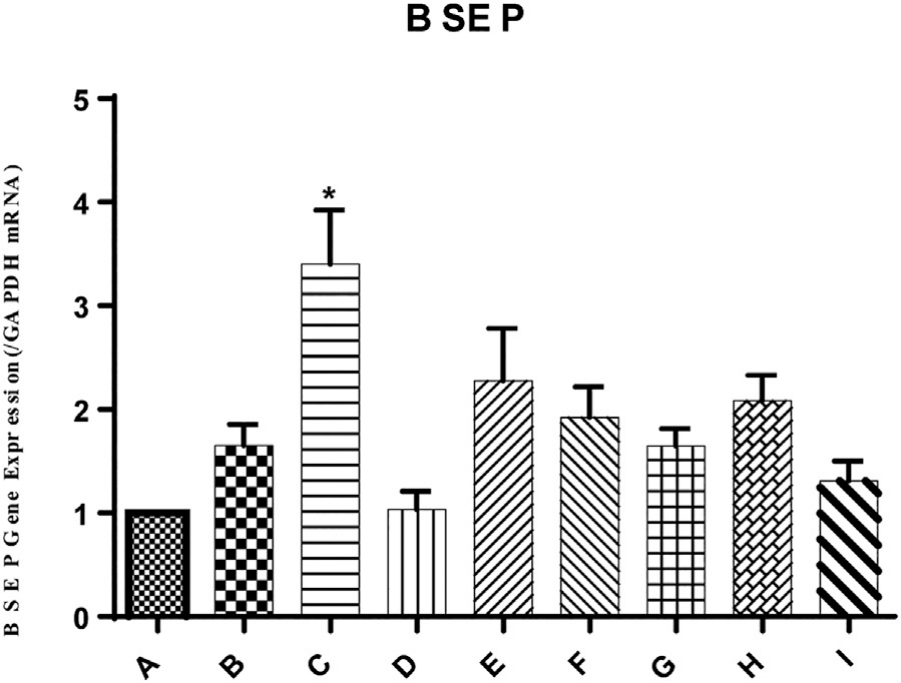
Effect of YGD on ANIT-treated cholestasis model mice. Mice were pretreated with different ratios of YGD (5 g/kg) or vehicle (olive oil) for 7 days prior to ANIT administration. Liver tissues were collected at 72 h after ANIT or vehicle administration. The mRNA expression level of BSEP was determined by RT-PCR. Normal (A), ANIT (B), ANIT + YGD (4:0) (C), ANIT + YGD (0:4) (D), ANIT + YGD (4:1) (E), ANIT + YGD (2:1) (F), ANIT + YGD (1:1) (G), ANIT + YGD (1:2) (H), and ANIT + YGD (1:4) (I). ^*^ *p* < 0.05 vs. normal group; ^#^ *p* < 0.05 vs. model group.

In the present study, the mRNA expression of the bile salt export pump (BSEP) in mouse liver was unchanged by ANIT group, as shown in Figure 4. Conversely, the mRNA expression level of BSEP in group C was 2.06-fold higher than that in the ANIT group. Similarly, the expression of BSEP in groups E and F was 1.38- and 1.16-fold higher than that in the ANIT group, while 1:1, 1:2, 1:4, and 0:4 YGD had no effect on mRNA expression of BSEP.

## Discussion

ANIT is a hepatotoxin experimentally used in rodents to model human intrahepatic cholestasis (Kossor et al., 1995). Excretion of ANIT into bile duct could damage the biliary epithelial cells (BEC) and induce BEC proliferation, leading to bile duct obstruction resulting from a high dose of ANIT treatment. Leakage of bile from destruction of BEC into the live can cause bile infarcts, and hepatocytes necrosis, and infiltration of neutrophils and monocytes around sinusoids when the bile duct was obstructed (Kodali et al., 2006; Cullen et al., 2016). In the present study, ANIT group mice showed a significant increase in the activities of plasma ALT (16.3-fold), AST (2.9-fold), ALP (3.0-fold), TBIL (22.2-fold), and TBA (17.3-fold) as compared with the normal group, signifying liver injury and cholestasis. Liver damages including cholangiocytes damage, hepatocyte necrosis, and inflammation cells infltration were observed in ANIT group mice. Pretreatment with 0:4 or 1:4 YGD resulted in a significant reduction in plasma activities of AST (62.8%, 68.9%), ALT (84.9%, 74.3%), ALP (67.5%, 77.9%), and TBIL (95.9%, 91.4%) as compared with those in ANIT group. Moreover, the liver TBA content returned to normal and the histological change were significantly improved as compared with those in ANIT group. It can be presumed that 0:4 and 1:4 YGD had a therapeutic effect on cholestasis caused by ANIT treatment. However, pretreatment with 4:0 or 4:1 YGD resulted in aggravation of ANIT-treated liver injury, as evidenced by an elevation in plasma TBIL, TBA, ALT, AST, and ALP activities, as well as liver histology.

In comparison with the normal group, the concentrations of liver TBA in ANIT group mice were significantly increased. Among these unconjugated and conjugated BAs, β-MCA, TCA and TMCA were substanially elevated and presented dominant percentage. Zhang et al. reported that the mixture of β-MCA, TCA and TMCA could induce proinflammtory mediators in hepotacytes (Zhang et al., 2012). In comparison with the ANIT group mice, TBA level was markedly decreased in the group D, H, or I. In contrast, intrahepatic cholestasis was worse in the group C than that in ANIT group, showing more evident characteristic foci of liver necrosis. These foci are thought to result from surrounding biliary tract rupture by ANIT treatment (Woolbright and Jaeschke, 2019). In the present study, we found that the mRNA expression of BSEP in mouse liver was significantly increased in the YGD 4:0 group as compared with that in ANIT group, suggesting that pretreatment with 4:0 YGD induced BSEP to increase BA-dependent bile flow and biliary pressure. Millimolar concentration of BAs can flow into the parenchyma *via* disrupted canals of Hering following ANIT induced bile duct obstruction. Previous *In vitro* experiments have suggested that BAs at 200–2000 μM can induce hepatocyte necrosis (Jansen et al., 2017). These results are consistent with our previous findings showing that UDCA aggravates liver injury in ANIT-treated model mice *via* the induction of BSEP (Zhang et al., 2018). Thus, induction of Yinchen to BSEP expression may be deleterious in obstructive cholestasis, such as PSC with segmental biliary obstruction (Roma et al., 2011; Zhu et al., 2015) and cholangiocarcinoma with bile duct blockage. However, our recent findings indicate that Yinchen can alleviate liver injury in 1% CA-induced cholestatic model mice *via* the induction of BSEP (data not shown), suggesting that Yinchen can induce BSEP to treat cholestasis when the bile duct is not obstructed.

Injury and necrosis of hepatocytes results in the release of sterile mediators referred to as damage-associated molecular patterns (DAMPs), such as high mobility group box 1 (HMGB1) and mitochondrial DNA (mtDNA) (Woolbright and Jaeschke, 2017). HMGB1, which localized to the nucleus, is passively released after injury and necrosis of hepatocytes and works as a necrosis signal for the immune system through cell-surface receptors. The DAMPs can bind to Toll-like receptor 4 (TLR4) expressed on Kupffer cells, which are resident macrophages of the liver, to induce downstream signals that lead to cytokine production and initiation of the inflammatory response (Woolbright and Jaeschke, 2019). This can promote the phosphorylation of cytoplasmic NF-κB and its translocation to the nucleus. The phosphorylated p65 and p50 form a dimer to initiate the production of proinflammatory cytokines, which is followed by the activation of the innate immune system and release of excessive inflammatory mediators (TNF-α, IL-1β, and IL-6) and chemokines (CCL2 and CXCL2). CCL2 and CXCL2 can promote the recruitment of monocytes and neutrophils to the hepatic sinus and sites of liver injury, leading to severe hepatic injury and massive liver necroses. TLR4, TLR2 and receptor for advanced glycation endproducts (RAGEs), expressing on the membrane of Kupffer cells, could promote the translocation of NF-κB to the nucleus, resulting in the production and release of pro-inflammatory cytokines and chemokines. Glycyrrhetinic acid, the main bioactive components in Gancao, had significant inhibition against LPS-induced TLR2 expression, while had no effect on RAGEs in RAW264.7 cells (data not shown). It could presumed that YGD (Yinchen:Gancao 0:4) might inhibit TLR2/NF-κB signal pathway to reduce the production of pro-inflammatory cytokines and chemokines.

**FIGURE 5:**
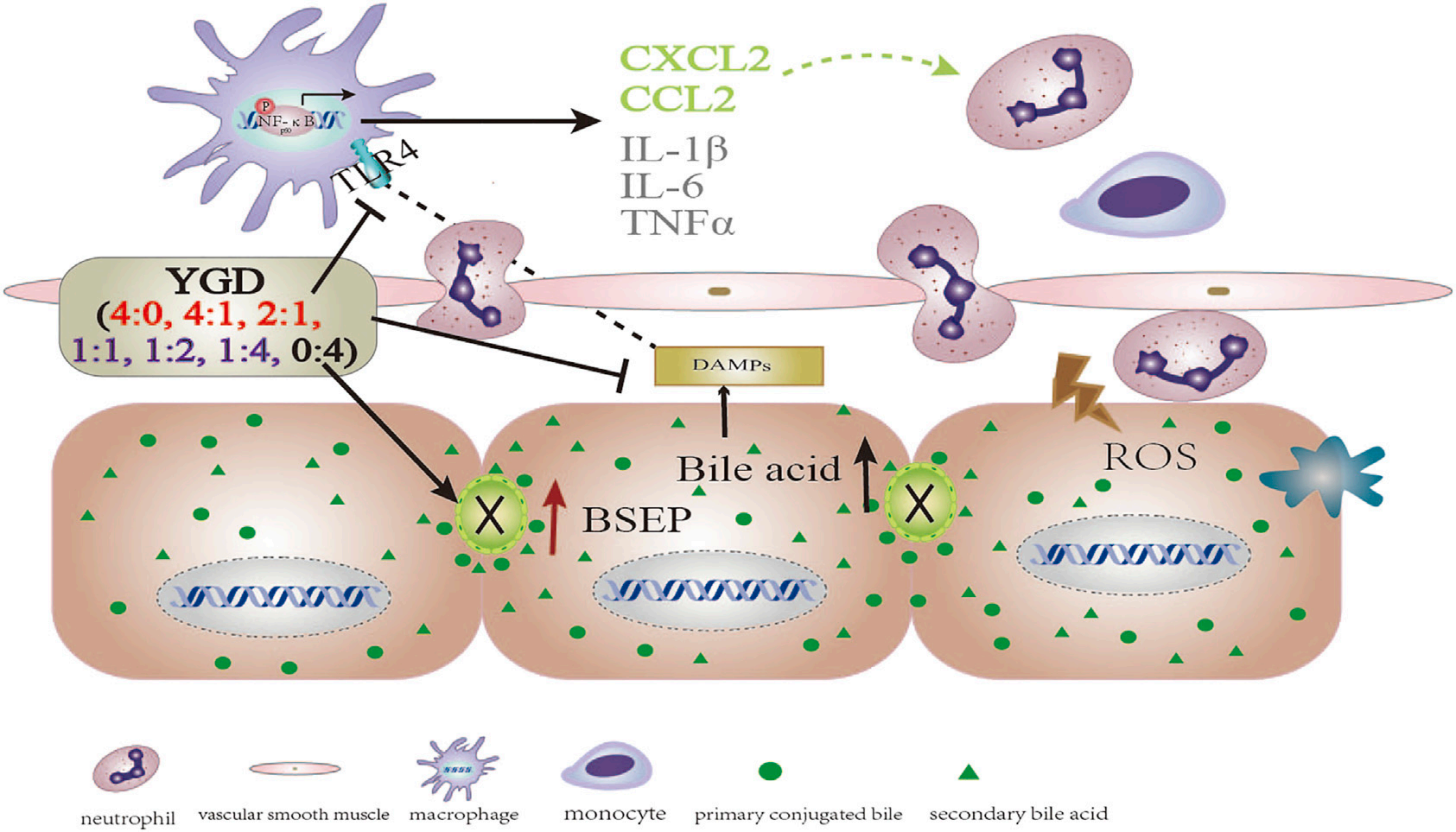
The mechanistic scheme for the effect of different YGD ratios on ANIT-treated cholestatic liver injury in mice.

In the present study, the hepatocyte expression of TLR4, MyD88, and p-NF-κBp65 and the levels of inflammatory cytokines (IL-1β, IL-6, and TNF-α) and chemokines (CCL2 and CXCL2) were increased in ANIT group mice. Treatment with 0:4 or 1:4 YGD markedly inhibited the expression of TLR4, MyD88, and p-NF-κBp65 and the levels of inflammatory cytokines and chemokines in the mouse liver. Therefore, YGD (1:4 and 0:4) treated cholestatic liver injury by inhibiting the TLR4/NF-κB signaling pathway and reducing the release of inflammatory cytokines and chemokines (Figure 5).

In summary, a high ratio of Gancao had a protective effect against ANIT-treated cholestatic liver injury in mice, the underlying mechanism of which may be attributed to the Gancao-mediated decrease in the hepatic accumulation of BA and its inhibition of the TLR4/NF-κB signaling pathway and inflammatory response. A high ratio of Yinchen had a minor protective effect against ANIT-treated cholestatic liver injury in mice, the underlying mechanism of which may be related to the Yinchen-mediated increase in BSEP levels and the reflux of BA due to bile duct obstruction (Figure 5).

According to the present study, Gancao can improve the inflammatory effect *via* inhibition of the TLR4/NF-κB pathway, while Yinchen can reduce hepatic accumulation of bile acids *via* induction of BSEP and increase bile acids excretion into bile duct. So, the combination of Yinchen and Gancao was beneficial to treating cholestasis without bile duct obstruction. Although our data obtained in mice could not be extrapolated to humans, cautions should be taken when a high dose of Yinchen was applied to patients with bile duct obstruction in clinical practice. In this situation, Gancao could be recommended to use. This information could be provide new insights into the effect of YGD in the treatment of cholestasis.

## Data Availability

The raw data supporting the conclusions of this article will be made available by the authors, without undue reservation.
